# Method comparison of Particle Enhanced Immunoturbidimetry (PEIT) with High Performance Liquid Chromatography (HPLC) for glycated hemoglobin (HbA1c) analysis

**DOI:** 10.1186/s40842-021-00123-w

**Published:** 2021-06-13

**Authors:** Shabnam Dildar, Sheharbano Imran, Farah Naz

**Affiliations:** 1 Department of Chemical Pathology, National Institute of Bloood Diseases and Bone Marrow Transplanataion, Karachi, Pakistan; 2 Section of Chemical Pathology, Department of Pathology, Fatima Memorial Medical and Dental College, Lahore, Pakistan; 3 Department of Pathology, Dr. Shamsi and Ansari Diagnostics Laboratory, Karachi, 74800 Pakistan

**Keywords:** HbA1c, Particle enhanced immunoturbidimetric test (PEIT), High performance liquid chromatography (HPLC)

## Abstract

**Background and objective:**

High Performance Liquid Chromatography (HPLC) technique is considered as a gold standard for HbA1c analysis however all laboratories cannot adopt it due to certain limitations. Our aim was to compare Particle Enhanced Immunoturbidimetry (PEIT) method with High Performance Liquid Chromatography (HPLC) for HbA1c analysis.

**Method:**

All blood samples were analyzed by HPLC assay on a Bio-Rad D-10 analyzer and PEIT on an Erba XL-200 analyzer. Precision studies were undertaken and Coefficient of Variation (%CV) calculated. Systemic Error (SE), Random Error (RE) and Total Error (TE_calc_) were obtained. The Total Allowable Error (TEa) set by the National Glycohemoglobin Standardization Program (NGSP) for HbA1c is 6%.The acceptable evaluation method is where TE_calc_ is less than TE_a._

**Results:**

The Precision studies were satisfactory with Coefficient of Variation (%CV) being less than 4% for both techniques. Mean HbA1c levels were slightly higher from HPLC than PEIT 9.07 ± 2.23% and 8.93 ± 2.10% respectively, although the difference was minimal. RE was 1.41%, TE_calc_ was 1.55%, which was less than TEa set by the NGSP. Both methods strongly correlated with the correlation coefficient (r) 0.9716, *p* < 0.0001.

**Conclusion:**

Our study showed HbA1c analysis by PEIT technique is precise, accurate, rapid and convenient and can be employed as an alternative to HPLC technique in countries where cost is a major problem for diagnosis and treatment.

## Introduction

Diabetes is highly prevalent in both developing and developed countries. National Diabetes Survey of Pakistan (NDSP 2016–2017) has reported the prevalence of diabetes as 26.3% in Pakistan [1]. The World Health Organization (WHO) has predicted a rise of 170% in the incidence of diabetes in developing countries [2]. HbA1c is considered as the most accurate, reliable and commonly used marker to assess blood glucose levels in the body for the past 3 months [3]. The DCCT (Diabetes Control and Complication Trial) group and some epidemiologists have found that the development and progression of micro vascular complications of diabetes are associated with long-term glycemic control [4, 5]. HbA1c test is recommended by the WHO and American Diabetes Association (ADA) for the diagnosis and monitoring of diabetes mellitus [2]*.*

Several analytical methods have been developed for HbA1c analysis. The most frequently used are ion-exchange chromatography and affinity chromatography for total glycated hemoglobin [6]. The National Glycohemoglobin Standardization Program (NGSP) improves the quality of HbA1c testing. ADA recommends that laboratories should use NGSP-certified methods for HbA1c analysis [7, 8]. Notwithstanding all these quality improvement measures, there is a degree of inter-method variability among NGSP-certified methods. Additionally, various laboratory methods are also available to measure HbA1c in blood. Many studies have revealed significant bias among analytical methods [5]. The allowable total error for HbA1c is 3.0% according to biological variation, while to 6.0% as per NGSP [9].

There is a need for standardization of HbA1c results with all available techniques. HPLC is a gold standard method for HbA1c analysis however, it is expensive, time consuming and requires technical skills which renders it difficult to adopt by every laboratory. The aim of this study was to perform the method comparison of the Particle Enhanced Immunoturbidimetry (PEIT) on an Erba XL-200 with High performance liquid chromatography (HPLC) and on a Bio-Rad-D10 analyzer for HbA1c analysis.

## Material and methods

Following from an institutional review committee approval, a cross sectional study was conducted from January 2019 to July 2019 at the Dr. Shamsi and Ansari Diagnostics Laboratory, Karachi, Pakistan.

Written informed consent was obtained from the participants. For Precision studies two levels of HbA1c control materials (high and normal levels) were run twenty times and mean, SD and Coefficient of Variation (%CV) was calculated. For method comparison study, three to five milliliters of blood samples were collected in EDTA tubes for HbA1c analysis.

The Total Error was calculated as TE_calc_ = bias_meas_ + 3s_meas_ [10–15]. The Systematic Error (SE) for HbA1c assays was calculated, for (X_c_) a medical decision of 6.5% was obtained and then the Y-value (Y_c_) was calculated. Systematic Error (SE) was calculated, after taking the difference between Y_c_ and X_c_. The Total Error was calculated as TE_calc_ = bias_meas_ + 3s_meas_ [10–15]. S_meas_ was the estimate from the replication experiment. The HbA1c assay is acceptable when Total Error (TE_calc_) is less than the Allowable Total Error (TE_a_). For method comparison patients of type 2 diabetes were involved.

### HbA1C techniques

#### PEIT technique on the Erba XL-200 HbA1c analyzer

In this technique, HbA1c was analyzed without measuring total hemoglobin. The measured absorbance of the HbA1c bound to particles was proportional to the percentage of HbA1c in the sample.

The technique is standardized according to IFCC reference method [16].This technique is NGSP certified and traceable to the DCCT reference method [8, 17].

#### HPLC technique on the Biorad-D10 HbA1c analyzer

The technique used was a chromatographic separation of the analytes by ion exchange HPLC. Hemoglobins were separated based on their ionic interactions. The absorbance was measured at 415 nm wavelength. A chromatogram was generated and the area of the HbA1c was calculated using an exponentially modified Gaussian (EMG) algorithm. This Technique is NGSP certified and traceable to the DCCT reference method [8, 17].

### Analysis

Data were entered in Microsoft Excel and then to the EP evaluator version 10.0 (statistical software) for analysis. HPLC method was considered as the gold standard or the X method while PEIT was considered as a test or the Y method. Mean, SD, and coefficient of variation (%CV) was calculated. Total Allowable Error was calculated. Percentage bias between two methods was calculated using Bland–Altman technique. The Correlation Coefficient (*r* value) was calculated and association of HPLC and PEIT was calculated. Scatter plots of test data and reference methods were created and their linear relationship was calculated using the deming regression model equation; (slope (b) and y-intercept (a)).

## Results

For the purpose of precision study two levels of controls (high and low) were run in a day and CV% values were calculated for both the Erba XL 200 analyzer and the Bio rad-D-10 analyzer as shown in Table 1. There was high precision found among both techniques and CV% was less than 4% as shown in Table 1 (below). Less than 5% CV is acceptable as per NGSP and IFCC.

**Table 1:** Precision study of HbA1c by both techniques

	**Method**	**Mean**	**Lower limit and upper limit**	**SD**	**CV%**
**Normal control HbA1c**	HPLC	5.4%	4.6–5.8%	0.21	3.8%
	PEIT	5.5%	4.1–6.0%	0.11	2.0%
**High control HbA1c**	HPLC	9.6%	8.8–10.0%	0.38	3.9%
	PEIT	9.7%	9.76–13.6%	0.23	2.3%

### Method comparison results

**Fig. 1:**
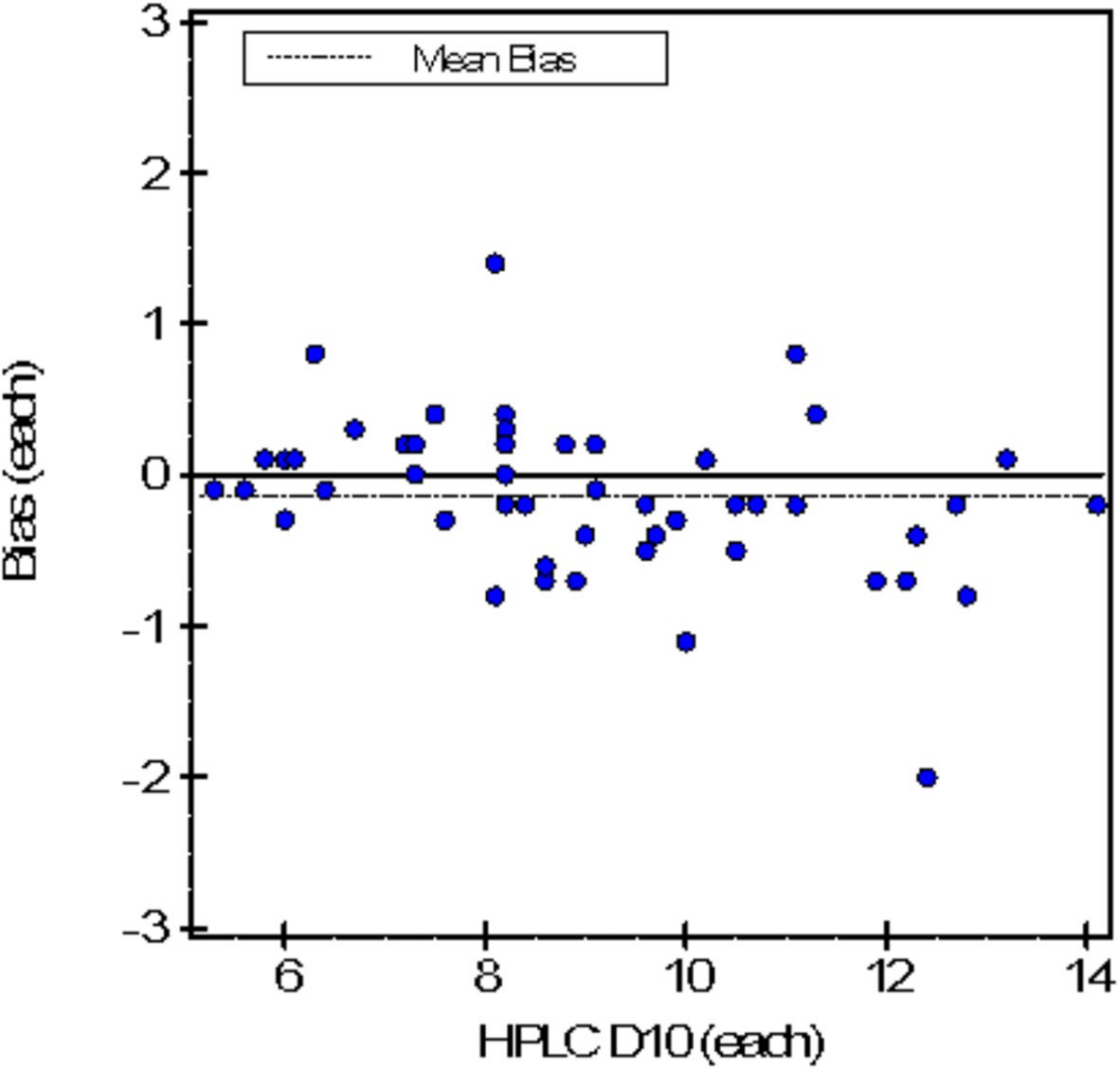
Bland–Altman plot of HPLCand PEIT

**Fig. 2:**
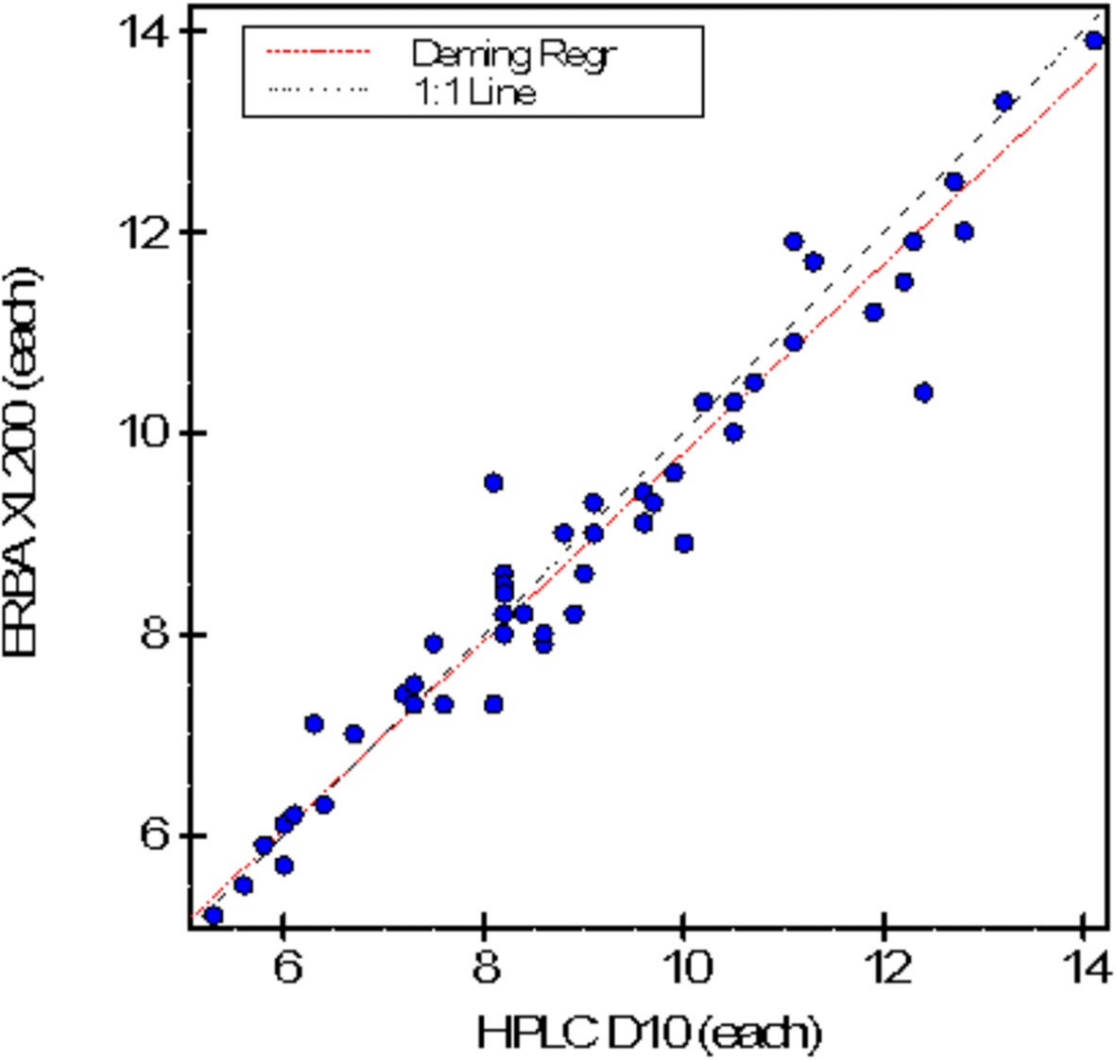
The Scatter plot of PEIT and HPLC

A total of 58 patients’ with type 2 diabetes were included, their samples were run by PEIT technique using the Erba XL 200 HbA1c analyzer and for the HPLC technique the Bio-rad-D10 HbA1c analyzer was used. The mean HbA1c value measured by HPLC method was 9.07 ± 2.23% and 8.93 ± 2.10% by PEIT method respectively. HbA1c values measured with the HPLC method were higher than those measured with PEIT and the results were statistically significant (*p* < 0.0001). The average bias was -0.14 (-1.56%), slope of 0.939 (95%CI 0.873–1.004) and intercept of 0.42 (95% CI-0.20–1.03) as shown in Fig. 1. TE_calc_ was 1.55%. The S_meas_ determined by the replication experiment. Method performance is acceptable when Total Error (TE_calc_) is less than the Allowable Total Error (TE_a_). There was positive concordance between results of both techniques with *r* = 0.9716, *p* < 0.0001 as shown in Fig. 2.

## Discussion

Glycated hemoglobin (HbA1C) test is not only used for the diagnosis but it is also used to adjust the dose and to assess the risk for the development of complications in diabetic patients. Presently, over a hundred methods exist for the analysis of HbA1c and some of them are traceable to DCCT. The reference method for HbA1c is HPLC however this is not affordable for all laboratories. In addition to this, it is time consuming, complex and requires trained technologists and expensive apparatus. Therefore, there is a dire need of other methods that are user friendly, cost effective and most importantly strongly correlated to the reference method. Although, great efforts have been made to improve the standardization of HbA1c methods no consensus exists on alternative methods.

Today, Immunoturbidimetric technique is widely used because it is efficient, cost effective and appropriate for countries with low/medium income population and allow rapid and reliable results. Furthermore, it is easy to perform and accessible in most developing countries.

Present study showed the method comparison of PEIT with HPLC to see its analytical performance for HbA1c analysis. We noted mean HbA1c level of patient samples slightly higher results with HPLC then PEIT, it may be due to fact that HPLC method measure the labile form of HbA1c (resulting from acute changes of blood glucose) producing a false increase in HbA1c levels, while PEIT is insensitive towards the labile factor of HbA1C. Sherwani et al. in 2016 also reported mean HbA1c measured by HPLC (7.52% ± 1.40%) was significantly higher than the PEIT (7.26% ± 1.39%), there was good concordance between results of PEITT and HPLC methods with *r* value of 0.94 [18]. The present study showed positive concordance with results of PEIT and HPLC methods with *r* = 0.97, *p* < 0.0001. Sabarinathan M et al. in 2020 estimated HbA1c levels in 50 diabetics patients by using same methods and showed a good positive correlation with *r* value of 0.992 [19]. Paolo Metus et al. also reported good correlation of PEIT and HPLC with *r* = 0.98, and this close agreement with the HPLC ion-exchange makes PEIT as highly specific and sufficiently precise assay for HbA1c analysis [20].

In present study the mean HbA1c value measured by HPLC (9.07 ± 2.23%) method were higher than those measured with PEIT (8.93 ± 2.10%) it may be due to fact that HPLC method measure the labile form of HbA1c (resulting from acute changes of blood glucose) producing a false increase in HbA1c levels, while PEIT is insensitive towards the labile factor of HbA1C [21]. The % CV of both techniques in present study was < 4%, which is good and in agreement with the recommended % CV between day < 5% [9, 22].

## Conclusion

The PEIT method is precise, rapid, easier to perform and ha high correlation with HPLC results and therefore it can be used as an alternative to HPLC methods in developing countries where cost is a major issue for diagnosis and treatment.

## Data Availability

Data is confidential.

## Abbreviations

HbA1c: Glycated hemoglobin
PEIT: Particle Enhanced Immunoturbidimetry
HPLC: High Performance Liquid Chromatography
SE: Systemic error
RE: Random error
TEa: Total allowable error
NGSP: National Glycohemoglobin Standardization Program
NDSP: National Diabetes Survey of Pakistan
WHO: World health organization
DCCT: Diabetes Control and Complication Trial
%CV: Coefficient of variation
